# Editor in Chief changes for *Implementation Science*

**DOI:** 10.1186/1748-5908-7-81

**Published:** 2012-08-31

**Authors:** Martin P Eccles

**Affiliations:** 1 Institute of Health and Society, Newcastle University, The Baddiley-Clark Building, Richardson Road, Newcastle upon Tyne, NE2 4AX, UK

## Abstract

Implementation Science, like all journals, needs to continue to develop. There will always be changes we need to make as next steps in improving the Journal for readers and improving how it runs. However, we now have our first change in Editors in Chief. We are fortunate to have been able to recruit two experienced academics who are also experienced editors—Professor Michel Wensing and Dr Anne Sales. I hope you will join me in welcoming them and give them, and continue to give *Implementation Science*, your support.

## 

Since November 2005, when *Implementation Science* first accepted submissions, and February 2006 when it published its first issue, the Journal has grown from 100 submissions in 2006 to 354 in 2011. Similarly, the number of articles published has also increased from 29 in 2006 to 133 in 2011.

As well as providing a high-quality, peer-reviewed outlet for traditional research articles, we have encouraged the publication of a range of articles that were previously difficult (if not impossible) to publish despite the widely held view that they were important contributions to the field—study protocols, detailed descriptions of barriers and facilitators to high-quality care, detailed descriptions of the development processes behind interventions, evaluations of the processes by which interventions achieved their effects—as well as providing space for conceptual and methodological articles.

The strong response to the Journal launch, and its year-on-year increase in submissions and publications, speak to the importance of the Journal to the emerging field of implementation science. Listed in all the major electronic databases, we attracted our first impact factor in 2010 and this has steadily risen to our current figure of 3.1. We are now ranked 4^th^ out of 62 journals in the ‘Health Care Sciences & Services’ and 9^th^ out of 76 journals in the ‘Health Policy & Services' categories of the Journal Citation Report. With our clear focus on implementation science, we are now the leading international journal in this field.

As we have grown, we have made changes to continue the efficient running of the Journal. We have recruited an increasing number of Associate Editors. We have gone from starting with just the two Co-Editors in Chief to now having one Deputy Editor in Chief and ten Associate Editors. This has been part of a steady, incremental process, and the Editors in Chief are enormously grateful to the Associate editors for the work that they contribute to running the Journal. We have re-configured our Editorial Board to form two bodies—a Senior Advisory Board to provide the Editor(s) in Chief with strategic advice when it is required and a refreshed and enlarged editorial board. We have a loyal pool of editorial board members who review for us on a regular basis, and we are very grateful for all of the time they commit to Journal activities.

And so to our next transition. Last year, Brian Mittman became Editor in Chief Emeritus, leaving me as sole Editor in Chief—a role that I will step down from during the next twelve months when I will have been Editor in Chief for over seven years. I am delighted to announce that, as of 1 September 2012, two of our current editors—Professor Michel Wensing and Dr Anne Sales—are taking on the roles of Co-Editors in Chief. They and I will share the Co-Editor in Chief roles until I permanently stand down in 2013.

*Implementation Science*, like all journals, needs to continue to develop. There are a number of changes we need to make as the next steps in improving the Journal for readers and improving how it runs. However, this change in Editors in Chief is key to the continuing development of the Journal. We are fortunate to be able to recruit two experienced academics who are also experienced editors, and it is good to be planning the transition with two such experienced pairs of hands. I hope you will join me in welcoming them and give them, and continue to give *Implementation Science,* your support.

